# Operative Surgery and Management

**Published:** 1987-11

**Authors:** M. G. Wilson


### Book Review

**OPERATIVE SURGERY AND MANAGEMENT**

**2nd Edition**

**G. Keen**

**Wright, Bristol. £65**

The first edition of this book appeared in 1981 since when there have been important advances in the practice of surgery, and now a second edition keeps it up to date. A number of important additions have been made including chapters on Microvascular surgery, the use of Lasers and of automatic stapling devices. There is also a special chapter on the phenomenally successful Shouldice repair of inguinal hernia from Toronto. The list of authors has increased from 54 to 61. Despite the addition of 7 chapters and 150 pages the new volume is smaller and more likely to fit your bookcase. Many of the chapters have been revised by their authors and provided with new illustrations. Basic considerations of anatomy and physiology, diagnostic procedures and indications for surgery are included as well as preparation for surgery and care afterwards. Although it deals with high technology this book is primarily addressed to general surgeons; the specialist sections, e.g. on gynaecology, have been written with them in mind. It will therefore be especially useful to general surgeons approaching unfamiliar territory, particularly to those working in isolation who have to tackle whatever problems present themselves and to whom such a book is a boon and a godsend.

Of Mr Keen's team of collaborators half are from the Bristol area, but they are supported by another third of U.K. surgeons and one sixth from the USA and Canada, an enormous undertaking and it is doubtful if a better team could have been assembled. This invaluable book will continue to help surgeons the world over and to recommend the centre of its origin.

M. G. Wilson

